# Demographics and Clinical and Endoscopic Characteristics of Patients with *Helicobacter pylori* Infection and Gastroesophageal Reflux Disease: A Case-Control Study

**DOI:** 10.1155/2019/3819893

**Published:** 2019-05-06

**Authors:** Amir Mari, Naim Mahroum, Nicola Luigi Bragazzi, Mahran Shalaata, Tawfik Khoury, Abdulla Watad, Mahmud Mahamid

**Affiliations:** ^1^Endoscopy Unit, Nazareth Hospital EMMS, 16100 Nazareth, Israel; ^2^Azrieli Faculty of Medicine, Bar-Ilan University, 13195 Safed, Israel; ^3^Department of Medicine, Sheba Medical Center, Tel-Hashomer, Israel; ^4^Sackler Faculty of Medicine, Tel Aviv University, 52621 Tel Aviv, Israel; ^5^School of Public Health, Department of Health Sciences, University of Genoa, 16132 Genoa, Italy; ^6^Internal Medicine Department, Nazareth Hospital EMMS, 16100 Nazareth, Israel; ^7^Institute of Gastroenterology and Liver Diseases, Naharya Medical Centre, Bar-Ilan University, 13195 Safed, Israel; ^8^Department of Medicine ‘B', Sheba Medical Center, Tel-Hashomer, Israel

## Abstract

**Background:**

The correlation between *Helicobacter pylori* (*H. pylori*) and gastroesophageal reflux disease (GERD) is complex. Some studies showed a protective role of *H. pylori* infection against GERD. This study was aimed at assessing the role of *H. pylori* infection in GERD utilizing a large cohort of patients diagnosed with GERD.

**Methods and Materials:**

All patients who underwent gastroscopy for an indication of GERD during the study period between 2015 and 2017 at the EMMS Nazareth Hospital were considered eligible for the study and therefore were included. *H. pylori* infection was determined by the rapid urease test or histology. Severity of esophagitis was assessed using the Los Angeles classification. Univariate analysis was performed to figure out differences between patients according to *H. pylori* infection status. Multivariate regression analysis was conducted to illustrate the predictors of positivity for *H. pylori* infection.

**Results:**

2,508 GERD patients were included with a median age of 49.42 ± 17.96 years. *H. pylori* infection was detected in 299 (11.9%) patients. GERD patients with *H. pylori* infection were found to be younger (48.83 ± 17.42 years versus 44.57 ± 17.69 years, *p* < 0.001), have the tendency to smoke more (1406 (63.6%) versus 266 (89.0%), *p* < 0.001), and use more proton pump inhibitors or PPIs (1314 (59.5%) versus 242 (80.9%), *p* < 0.001). In multivariate regression analysis, age (OR 0.987, *p* < 0.001), smoking status (OR 0.190, *p* < 0.001), use of ASA/NSAIDs (OR 1.652, *p* = 0.004), and use of statins (OR 0.499 (95%CI 0.295-0.846), *p* = 0.010) were found significant among *H. pylori*-positive individuals. *H. pylori*-positive subjects have less severe esophagitis and more hiatus hernia.

**Conclusion:**

Patients with GERD and *H. pylori* infection were significantly younger, tended to smoke more, and used more PPIs and had significantly lower grades of esophagitis compared to noninfected ones.

## 1. Introduction

The association between infectious agents and gastritis has been known for centuries. However, this relation was not strongly implicated until the discovery of *Helicobacter pylori* (*H. pylori*) in the early 1980s [1]. Since then, and in addition to gastritis, strong evidences have been accumulated regarding the role of *H. pylori* in multiple gastric disorders including peptic ulcer disease [2, 3], gastric cancer, and lymphoma [4, 5]. *H. pylori* is spiral-shape slow-growing gram-negative bacteria characterized by urease, catalase, and oxidase positivity [6]. Urease activity is crucial for bacterial survival, as well as constituting the basis of *H. pylori* diagnostic testing [7]. In developed countries, the *H. pylori* infection rate is low in childhood; however, it increases with age reaching 10 percent in adulthood and raising up to 50 percent in individuals older than 60 years [8, 9]. Low socioeconomic status, overcrowding, smoking, family member with *H. pylori*, and unfiltered drinking water were all shown to be directly associated with increased prevalence rates [10, 11]. The isolation of *H. pylori* from gastric secretions as well as stool culture explains transmissibility among family members [12]. Diagnostic tests of infection are classified as invasive and noninvasive and are based mainly on urease activity. Invasive tests include the urease test, culture, histology, and polymerase chain reaction (PCR) in which gastroscopy is required to obtain stomach tissue [7, 13, 14]. *H. pylori* infection, if not properly treated, leads to the insurgence of many digestive disorders, such as chronic gastritis, peptic ulcer, upper gastrointestinal bleeding, MALToma, chronic atrophic gastritis, intestinal metaplasia, and distal gastric adenocarcinoma, among others. Triple or quadruple antibiotic-based regimens are the mainstay of therapy. Detailed guidelines for the treatment of *H. pylori* infection were released by the American College of Gastroenterology (ACG) and the Maastricht Group [15, 16]. *H. pylori* and GERD are common gastrointestinal conditions in the general population and may coexist. The relationship between *H. pylori* and GERD symptoms and esophagitis is still controversial. Despite the fact that numerous papers showed a protective role of *H. pylori* against GERD and esophagitis [17, 18], one landmark meta-analysis showed inconsistent results [19]. The suggestive protective role has been related to decreased gastric acid secretion due to atrophic gastritis in patients infected with *H. pylori* [18]. Moreover, reports showed that eradication of *H. pylori* by restoring gastric acid secretion could exacerbate GERD symptoms [20]. Taking these controversies into consideration, we aimed in our study to characterize demographics and endoscopic findings in patients with GERD according to their *H. pylori* infection status.

## 2. Material and Methods

### 2.1. Ethical Clearance

The current study received ethical approval from the local hospital ethical committee and was conducted according to the 1964 Helsinki declaration and its subsequent amendments. The data was coded in order to preserve the anonymity of the patients. Informed consent was waived because of the noninterventional design of the study.

### 2.2. Patient Selection

All patients referred to the endoscopy unit at the EMMS Nazareth Hospital (a district university-affiliated hospital, Nazareth, Israel) for esophagogastroduodenoscopy (EGD) due to GERD symptoms such as heartburn and/or regurgitations or refractory GERD, typical symptoms, or the presence of alarm symptoms between the years 2015 and 2017 were considered potentially eligible and enrolled in the study. Patients were allocated retrospectively, through electronic medical reports at our unit software. All EGD procedures were performed at the single endoscopic unit, at the Nazareth Hospital, by five different senior gastroenterologists. Severity of esophagitis was assessed using the Los Angeles classification [21]. *H. pylori* infection was determined either by histological examination through a biopsy taken during the EGD or by the rapid urease test using commercially available kits in the unit (PyloPlis+ manufactured by Gulf Coast Medical Inc., Oldsmar, FL 34677, USA).

### 2.3. Statistical Analysis

Before proceeding with any statistical handling and processing, data were visually inspected for potential outliers. The assumption of normal distribution of data was checked conducting the D'Agostino-Pearson omnibus test. Continuous data were expressed as means and standard deviations, whereas categorical parameters were computed as percentages, where appropriate.

Univariate analysis (chi-squared test, Student's *t*-test, and Kruskal-Wallis test) was performed to capture the main differences between subjects seronegative and seropositive for *H. pylori*.

Multivariate logistic or Poisson's regression analysis was conducted to shed light on the predictors of positivity for *H. pylori*, adjusting for potential confounders.

All statistical analyses were performed with the commercial software “Statistical Package for the Social Sciences” (SPSS version 24.0, IBM, Chicago, IL, USA). A *p* value of less than 0.05 was considered statistically significant.

## 3. Results

A sample of 2,508 subjects with classical clinical GERD symptoms of heartburn and regurgitations, aged 49.42 ± 17.96 years, was included in the study: most were Arabs (83.7%), 52.4% were females, 66.7% were current smokers, and 10.3% had comorbidities. Concerning pharmacological treatment, 62.0% consumed PPIs, whereas 7.6% used statins. *H. pylori* infection was found positive in 299 patients (11.9%). For further details, the reader is referred to Table 1.

In the univariate analysis (Table 2), *H. pylori*-negative and *H. pylori*-positive patients differed for age (48.83 ± 17.42 years versus 44.57 ± 17.69 years, *p* ≤ 0.001), smoking status (1406 (63.6%) versus 266 (89.0%), *p* > 0.001), and use of PPI (1314 (59.5%) versus 242 (80.9%), *p* ≤ 0.001), respectively. 70.6% of the *H. pylori*-non-infected group had no signs of esophagitis in endoscopy. 29.4% had esophagitis in the following LA classification severity order (17.2% had esophagitis LA A, 9.8% had esophagitis LA B, and 1.9% and 0.5% had LA D). In turn, 54.1% of the *H. pylori*-infected group had signs of esophagitis in endoscopy, in the following LA classification severity order (33.1% had esophagitis LA A, 9.7% had esophagitis LA B, 1.9% and 1.3% had LA C, and 0% had LA D). *H. pylori*-infected patients had less hiatus hernias (21.7%), compared to noninfected patients (30.7%).

In the multivariate logistic regression analysis (Table 3), age (OR 0.987 (95%CI 0.980-0.994), *p* < 0.001), smoking status (OR 0.190 (95%CI 0.112-0.324), *p* < 0.001), use of aspirin/nonsteroidal anti-inflammatory drugs (ASA/NSAIDs) (OR 1.652 (95%CI 1.169-2.333), *p* = 0.004), and use of statins (OR 0.499 (95%CI 0.295-0.846), *p* = 0.010) were assessed.

Concerning the outcomes, *H. pylori*-infected and noninfected subjects differed in terms of insurgence (*p* < 0.001) and severity (*p* = 0.003) of esophagitis (Table 4(a)) and insurgence of hiatal hernia (*p* = 0.001, Table 4(b)). No differences could be found in terms of prevalence of Barrett's esophagus.

Confirming the univariate analysis, the multivariate logistic regression shows that with respect to *H. pylori*-positive GERD patients, *H. pylori*-negative GERD individuals have a crude odds ratio (OR) of 1.598 (95%CI 1.196-2.134, *p* = 0.002) and a crude OR of 1.641 (95%CI 1.036-2.600, *p* = 0.035) of developing hiatal hernia (Table 5). Conversely, they had a crude OR of 0.527 (95%CI 0.412-0.675, *p* < 0.001) and an adjusted OR of 0.611 (95%CI 0.474-0.787, *p* < 0.001) of developing esophagitis (Table 6). Positivity for *H. pylori* impacted also on the severity of esophagitis (*p* = 0.047, Table 7).

## 4. Discussion

During the last decade, there has been a decrease in the prevalence of *H. pylori* infection; the prevalence of GERD, Barrett's esophagus, and esophageal adenocarcinoma has increased [22]. In our study, the prevalence of *H. pylori* infection among patients with symptoms suggestive of GERD was approximately 12%, which goes in parallel with the assumed inverse correlation between these two conditions. Chung et al. [23] showed in a Korean cohort of matched age and sex individuals lower prevalence of *H. pylori* infection in patients with GERD compared to controls. Furthermore, the authors reported that this association is negatively maintained throughout advanced grades of esophagitis, therefore suggesting a protective role of *H. pylori* against GERD. Indeed, our study found similar findings showing that advanced stages of esophagitis are less prevalent in patients with GERD and with *H. pylori* infection.

Epidemiological studies worldwide showed *H. pylori* infection to be acquired earlier in life in developing countries in opposite to developed countries where infection peaks after the age of 60 [24]. The median age of patients with GERD enrolled in our study differed among *H. pylori*-infected and noninfected individuals. *H. pylori*-infected patients were much younger than noninfected persons indicating an earlier acquisition of infection. Our findings suggest closer pictures to developing world. In terms of ethnicity, in contrast to the findings of Everhart et al. [25] that Blacks and Hispanics were found to have a higher prevalence of *H. pylori* infection, we found no difference in both groups analyzed. The authors concluded this ethnic variation to be probably explained by differences in socioeconomic status.

Relationship between smoking status and *H. pylori* infection has given contrasting findings in the literature [26]. Ogihara and collaborators found that current smokers had a 0.82- (95%CI 0.74-0.91) fold greater risk of *H. pylori* infection than those who had never smoked, with current cigarette consumption exhibiting a dose-dependently negative association with *H. pylori* [27]. This association was strong in younger subjects. Authors postulated that smoking-induced increased gastric acidity could play a major role in leading to *H. pylori* infection. Furthermore, polymorphisms could explain the relationship between smoking status and *H. pylori* [28]. On the other hand, Zhang and coauthors failed to find a statistically significant relationship between smoking status and *H. pylori* infection. Our data showed significantly higher smoking rates among *H. pylori*-positive individuals compared to negative persons [29].

In our study, alcohol drinking was not associated with *H. pylori* infection. In other studies, such as that by Ogihara and coworkers, current drinkers had a 0.88- (95%CI 0.79-0.98) fold greater risk of *H. pylori* infection than those who had never consumed alcohol [28].

Giral et al. [23] showed that PPI use in patients infected with *H. pylori* was associated with greater inhibition of gastric acid secretion when compared to uninfected individuals. Similar findings were demonstrated by Verdu et al.; by this manner, our data showed that *H. pylori*-infected patients have significantly greater proportion of PPI use. These findings have two main implications. First, while PPI is a guideline-recommended part of therapy, when it is used on top of *H. pylori* infection, it leads to substantially higher degrees of acid secretion inhibition. Second, while inhibition of gastric acid secretion seems to explain the protective role of *H. pylori* infection against GERD and higher degrees of esophagitis, this explains the lower prevalence rate of advanced grades of esophagitis in *H. pylori*-infected individuals. As shown in this, we saw our findings as going consistent with previous studies in medical literature.

While infection of *H. pylori* is assumed to be protective against GERD, the presence of hiatal hernia after eradication of *H. pylori* contributes to the development of GERD [30]. In our study, most of the patients enrolled had no hiatal hernia. Taking into consideration the presence of symptoms of GERD in all patients enrolled, hiatal hernia was more common in *H. pylori*-negative patients. This sheds light on the pathogenetic correlation between GERD and *H. pylori* in the presence of hiatal hernia.

Our study, when compared to similar studies in the literature, has several strong points: a large cohort of patients with well-defined characteristics, as well as a quite uniform ethnic homogeneity, as this prevents possible bias. Another point is that patients were screened endoscopically due to GERD symptoms including not only those with esophagitis but also those who were previously diagnosed with GERD as this permitted a larger cohort to be included.

The retrospective manner of the study and operator-dependent endoscopy and the clinical diagnosis of GERD without having PH monitoring to confirm acid reflux are all part of our limitations.

In conclusion, patients with GERD who tested positive for *H. pylori* were younger, tend to smoke more, and use more PPI than those who tested negative. In addition, patients infected with *H. pylori* had more esophagitis; however, they had less severe esophagitis compared to noninfected patients.

## Figures and Tables

**Table 1:** Descriptive statistics of the sample studied.

Parameter	Value (*n* = 2,508)
Ethnicity	
Arabs	2,100 (83.7%)
Jews	408 (16.3%)
BMI	29.67 ± 7.02, 29.0
Sociodemographic status	
Rural	1,524 (60.8%)
Urban	984 (39.2%)
Age	49.42 ± 17.96, 50.0
Gender	
Males	1,194 (47.6%)
Females	1,314 (52.4%)
Smoking status	1,672 (66.7%)
Alcohol consumption	96 (3.8%)
Comorbidities	259 (10.3%)
ASA/NSAID users	680 (27.1%)
Statin users	190 (7.6%)
PPI users	1,556 (62.0%)

ASA: acetylsalicylic acid; NSAIDs: nonsteroidal anti-inflammatory drugs; PPIs: proton pump inhibitors.

**Table 2:** Values broken down according to *Helicobacter pylori* infection status (univariate analysis).

Parameter	Subjects without *H. pylori* infection (*n* = 2,209)	Subjects with *H. pylori* infection (*n* = 299)	*p* value
Ethnicity			0.157
Arabs (*n* = 2,100)	1,841 (83.3% of *H. pylori* subjects, 87.7% of Arabs)	259 (86.6% of *H. pylori* subjects, 12.3% of Arabs)	
Jews (*n* = 408)	368 (16.7% of *H. pylori* subjects, 90.2% of Jews)	40 (13.4% of *H. pylori* subjects, 9.8% of Jews)	
BMI	29.69 ± 7.67	29.91 ± 6.29	0.297
Sociodemographic status			0.256
Rural (*n* = 1,524)	1,333 (60.3% of *H. pylori* subjects, 87.5% of rural subjects)	191 (63.9% of *H. pylori* subjects, 12.5% of rural subjects)	
Urban (*n* = 984)	876 (39.7% of *H. pylori* subjects, 89.0% of urban subjects)	108 (36.1% of *H. pylori* subjects, 11.0% of urban subjects)	
Age	48.83 ± 17.42	44.57 ± 17.69	*p* ≤ 0.001
Gender			0.902
Males (*n* = 1,194)	1,053 (47.7% of *H. pylori* subjects, 88.2% of males)	141 (47.2% of *H. pylori* subjects, 11.8% of males)	
Females (*n* = 1,314)	1,156 (52.3% of *H. pylori* subjects, 88.0% of females)	158 (52.8% of *H. pylori* subjects, 12.0% of females)	
Smoking status (*n* = 1,672)	1,406 (63.6% of *H. pylori* subjects, 84.1% of smokers)	266 (89.0% of *H. pylori* subjects, 15.9% of smokers)	*p* ≤ 0.001
Alcohol consumption (*n* = 96)	82 (3.7% of *H. pylori* subjects, 85.4% of alcohol consumers)	14 (4.7% of *H. pylori* subjects, 14.6% of alcohol consumers)	0.421
Comorbidities (*n* = 259)	222 (10.0% of *H. pylori* subjects, 85.7% of subjects with comorbidities)	37 (12.4% of *H. pylori* subjects, 14.3% of subjects with comorbidities)	0.224
ASA/NSAID users (*n* = 680)	613 (27.8% of *H. pylori* subjects, 90.1% of ASA/NSAID users)	67 (22.4% of *H. pylori* subjects, 9.9% of ASA/NSAID users)	0.052
Statin users (*n* = 190)	164 (7.4% of *H. pylori* subjects, 86.3% of statin users)	26 (8.7% of *H. pylori* subjects, 13.7% of statin users)	0.416
PPI users (*n* = 1,556)	1,314 (59.5% of *H. pylori* subjects, 84.4% of PPI users)	242 (80.9% of *H. pylori* subjects, 15.6% of PPI users)	*p* ≤ 0.001

ASA: acetylsalicylic acid; BMI: body mass index; NSAIDs: nonsteroidal anti-inflammatory drugs; PPIs: proton pump inhibitors.

**Table 3:** Predictors of *Helicobacter pylori* infection (multivariate logistic regression analysis).

Parameter	OR	95%CI OR		
		Lower bound	Upper bound	*p* value
Ethnicity (Arabs versus Jews)	1.105	0.765	1.595	0.596
BMI	1.002	0.988	1.017	0.747
Rural versus urban	1.144	0.883	1.481	0.308
Age	0.987	0.980	0.994	0.001
Gender	1.008	0.786	1.293	0.951
Smoking status	0.190	0.112	0.324	0.001
Alcohol consumption	0.773	0.425	1.406	0.398
Comorbidities	0.781	0.534	1.143	0.203
ASA/NSAID users	1.652	1.169	2.333	0.004
Statin users	0.499	0.295	0.846	0.010
PPI users	1.116	0.716	1.741	0.627
Constant	0.567			0.301

ASA: acetylsalicylic acid; BMI: body mass index; NSAIDs: nonsteroidal anti-inflammatory drugs; PPIs: proton pump inhibitors.

**Table:** (a) Endoscopic findings of GERD (insurgence and severity of esophagitis) subjects stratified according to the *Helicobacter pylori* (*H. pylori*) infection status

*H. pylori* status (*n* = 2.508)	No esophagitis (*n* = 1,726)	Esophagitis (*n* = 782)			
		Grade A (*n* = 478)	Grade B (*n* = 245)	Grade C (*n* = 47)	Grade D (*n* = 12)
*H. pylori* negative (*n* = 2,209)	1,559 (70.6%)	379 (17.2%)	216 (9.8%)	43 (1.9%)	12 (0.5%)
*H. pylori* positive (*n* = 299)	167 (55.9%)	99 (33.1%)	29 (9.7%)	4 (1.3%)	0 (0.0%)

**Table:** (b) Outcomes of subjects with GERD symptoms (insurgence of hiatal hernia) stratified according to their *Helicobacter pylori* infection status

*H. pylori* status (*n* = 2,508)	No hiatal hernia (*n* = 1,764)	Hiatal hernia (*n* = 744)

*H. pylori* negative (*n* = 2,209)	1,530 (69.3% of *H. pylori*-negative subjects, 86.7% of subjects without hiatal hernia)	679 (30.7% of *H. pylori*-negative subjects, 91.3% of subjects with hiatal hernia)
*H. pylori* positive (*n* = 299)	234 (78.3% of *H. pylori*-positive subjects, 13.3% of subjects without hiatal hernia)	65 (21.7% of *H. pylori*-positive subjects, 8.7% of subjects with hiatal hernia)

**Table 5:** Determinants of insurgence of hiatal hernia in GERD patients (multivariate logistic regression analysis).

Parameter	OR	95%CI OR		
		Lower bound	Upper bound	*p* value
*H. pylori* status (negative versus positive)	1.641	1.036	2.600	0.035
Ethnicity (Arabs versus Jews)	0.993	0.685	1.441	0.972
BMI	1.000	0.982	1.018	0.968
Rural versus urban	0.768	0.583	1.012	0.061
Age	1.014	1.006	1.022	0.001
Gender	0.950	0.724	1.248	0.713
Smoking status	0.916	0.539	1.555	0.745
Alcohol consumption	0.955	0.480	1.903	0.897
Comorbidities	0.730	0.484	1.102	0.134
ASA/NSAID users	0.001	0.001	0.002	*p* ≤ 0.001
Statin users	253.885	89.148	723.041	*p* ≤ 0.001
PPI users	0.591	0.346	1.007	0.053
Constant	0.334			0.074

ASA: acetylsalicylic acid; BMI: body mass index; NSAIDs: nonsteroidal anti-inflammatory drugs; PPIs: proton pump inhibitors.

**Table 6:** Determinants of insurgence of esophagitis in GERD patients (multivariate logistic regression analysis).

Parameter	OR	95%CI OR		
		Lower bound	Upper bound	*p* value
*H. pylori* status (negative versus positive)	0.611	0.474	0.787	*p* ≤ 0.001
Ethnicity (Arabs versus Jews)	0.786	0.622	0.995	0.045
BMI	0.995	0.982	1.007	0.395
Rural versus urban	0.957	0.801	1.143	0.629
Age	0.997	0.992	1.002	0.242
Gender	1.199	1.007	1.426	0.041
Smoking status	0.370	0.265	0.519	*p* ≤ 0.001
Alcohol consumption	0.859	0.551	1.339	0.503
Comorbidities	0.768	0.584	1.011	0.060
ASA/NSAID users	0.779	0.629	0.965	0.022
Statin users	1.209	0.831	1.761	0.321
PPI users	1.359	0.990	1.866	0.058
Constant	1.913			0.120

ASA: acetylsalicylic acid; BMI: body mass index; NSAIDs: nonsteroidal anti-inflammatory drugs; PPIs: proton pump inhibitors.

**Table 7:** Determinants of severity of esophagitis in GERD patients (Poisson's regression analysis).

Parameter	*B*	SE	Wald's chi-square	Sig.
Constant	0.027	0.2868	0.009	0.925
Ethnicity (Arabs versus Jews)	0.082	0.0799	1.062	0.303
Rural versus urban	−0.012	0.0607	0.039	0.843
Gender	0.002	0.0596	0.001	0.971
Smoking status	0.036	0.1146	0.096	0.756
Alcohol consumption	0.018	0.1494	0.015	0.902
Comorbidities	0.041	0.0919	0.202	0.653
ASA/NSAID users	−0.018	0.0710	0.067	0.796
Statin users	0.028	0.1285	0.046	0.830
PPI users	−0.086	0.1038	0.693	0.405
*H. pylori* status (negative versus positive)	0.168	0.0847	3.956	0.047
BMI	0.001	0.0048	0.001	0.996
Age	0.002	0.0017	1.779	0.182

ASA: acetylsalicylic acid; BMI: body mass index; NSAIDs: nonsteroidal anti-inflammatory drugs; PPIs: proton pump inhibitors.

## Data Availability

The data used to support the findings of this study are available from the corresponding author upon request. Data was collected retrospectively by authorized researchers.
